# Facilitators and barriers to community pharmacy PrEP delivery: a scoping review

**DOI:** 10.1002/jia2.26232

**Published:** 2024-03-17

**Authors:** China Harrison, Hannah Family, Joanna Kesten, Sarah Denford, Anne Scott, Sarah Dawson, Jenny Scott, Caroline Sabin, Joanna Copping, Lindsey Harryman, Sarah Cochrane, Jeremy Horwood

**Affiliations:** ^1^ National Institute for Health and Care Research Applied Research Collaboration West (NIHR ARC West) Bristol UK; ^2^ National Institute for Health and Care Research Health Protection Research Unit (HPRU) in Behavioural Science and Evaluation, Population Health Sciences, Bristol Medical School University of Bristol Bristol UK; ^3^ Population Health Sciences Bristol Medical School University of Bristol Bristol UK; ^4^ Centre for Academic Primary Care (CAPC) Bristol Medical School University of Bristol Bristol UK; ^5^ Institute for Global Health UCL Royal Free Campus London UK; ^6^ NIHR HPRU in Blood‐Borne and Sexually Transmitted Infections at UCL London UK; ^7^ Communities and Public Health Bristol City Council College Green Bristol Bristol UK; ^8^ Unity Sexual Health University Hospitals Bristol and Weston NHS Foundation Trust Bristol UK; ^9^ The Riverside Clinic Royal United Hospitals Bath NHS Foundation Trust Bristol UK

**Keywords:** PrEP, HIV, community pharmacy, COM‐B, barriers and facilitators, HIV prevention

## Abstract

**Introduction:**

Pre‐exposure prophylaxis (PrEP) is an effective medication to reduce the risk of acquiring HIV. PrEP is available free of charge in the UK from sexual health clinics. Expanding PrEP delivery to community pharmacies holds promise and aligns with UK government goals to eliminate new cases of HIV by 2030. The aim of this scoping review was to describe the existing evidence about the barriers to and facilitators of community pharmacy oral PrEP delivery, for pharmacists and pharmacy clients, as aligned with the Capacity Opportunity, Motivation Behaviour (COM‐B) Model.

**Methods:**

Five bibliographic and five review databases were searched from inception to August 2023. Literature of any study design was included if it discussed barriers and facilitators of community pharmacy PrEP delivery. Trial registrations, protocols and news articles were excluded.

**Results:**

A total of 649 records were identified, 73 full texts were reviewed and 56 met the inclusion criteria, predominantly from high‐income/westernized settings. Most of the included literature was original research (55%), from the United States (77%) conducted during or after the year 2020 (63%). Barriers to PrEP delivery for pharmacists included lack of knowledge, training and skills (capability), not having the necessary facilities (opportunity), concern about the costs of PrEP and believing that PrEP use could lead to risk behaviours and sexually transmitted infections (motivation). Facilitators included staff training (capability), time, the right facilities (opportunity), believing PrEP could be a source of profit and could reduce new HIV acquisitions (motivation). For clients, barriers included a lack of PrEP awareness (capability), pharmacy facilities (opportunity) and not considering pharmacists as healthcare providers (motivation). Facilitators included awareness of PrEP and pharmacist's training to deliver it (capability), the accessibility of pharmacies (opportunity) and having an interest in PrEP (motivation).

**Discussion:**

To effectively enhance oral PrEP delivery in UK community pharmacies, the identified barriers and facilitators should be explored for UK relevance, addressed and leveraged at the pharmacy team, client and care pathway level.

**Conclusions:**

By comprehensively considering all aspects of the COM‐B framework, community pharmacies could become crucial providers in expanding PrEP accessibility, contributing significantly to HIV prevention efforts.

## 1 INTRODUCTION

The human immunodeficiency virus (HIV) epidemic remains a significant public health concern worldwide. In 2021, it was estimated that there were 105,200 people living with HIV in the UK, approximately 2955 of whom had been newly diagnosed in that year [1, 2].

In recent years, pre‐exposure prophylaxis (PrEP) has emerged as an effective preventative biomedical intervention which reduces the risk of acquiring HIV by 92−99% [3−7]. PrEP can be taken orally daily or “on demand” before and after sex. To initiate PrEP, eligible clients are required to have baseline and regular health checks, including kidney function tests and sexually transmitted infections (STIs) screening, to ensure safety [8]. While internationally PrEP is available via pharmacies in some countries, in the UK, it is only available free of charge via National Health Service (NHS) sexual health clinics.

Disproportionately low uptake has been observed in some regions and among some populations at elevated risk of acquiring HIV, such as trans individuals [9], cisgender women [10, 11], young people [12] and people of Black African or Caribbean origin [13, 14]. Low uptake has been attributed to several barriers in accessing PrEP, including the geographical proximity of PrEP providers, stigma and lack of client awareness of PrEP [15]. Approximately 83% of people in need of PrEP had their need identified during a clinical consultation in 2022 highlighting the need to raise awareness of PrEP to support individuals’ treatment‐seeking autonomy [1, 15]. Expanding PrEP delivery to community pharmacies could result in raised awareness, reduced barriers to access, enhanced autonomy and increased utilization and coverage of PrEP [2, 7].

There are approximately 11,000 pharmacies in the UK distributed across urban/rural and deprived/affluent areas; 90% of people in England, and 98% of people in areas of highest deprivation are just 20 minutes’ walk away from their nearest community pharmacy with 90% of the population making at least one visit to the pharmacy per year [16]. Community pharmacies play a crucial role in delivering public health services and have expanding roles in health promotion and prescribing [17, 18]. Predominantly they are private businesses where pharmacy teams use their expert knowledge to clinically screen and dispense prescriptions, sell or supply over‐the‐counter medicines, give advice, administer vaccinations and deliver locally commissioned healthcare services. Their location, accessibility, convenience and customer rapport make them well‐suited to address urgent and preventative care needs [19, 20]. PrEP delivery could align well with existing service provision within pharmacies with pharmacy teams routinely dispensing prescriptions for sexual and reproductive health medicines (e.g. emergency hormonal contraception). Further, pharmacy‐based interventions have demonstrated success in improving medication adherence for various medications including PrEP [21]. For example, in the United States, the implementation of the One Step PrEP programme at a Seattle pharmacy enabled pharmacists to prescribe and manage PrEP resulting in a 90% PrEP adherence rate among attendees with two or more visits [22]. While implementing community pharmacy PrEP delivery in the UK could benefit individuals at risk of acquiring HIV, community pharmacy PrEP delivery would require training, support and a behavioural change by pharmacists and people (hereafter, clients) attending their services.

The Capability Opportunity Motivation Behaviour (COM‐B) model offers a valuable framework to understand the barriers and facilitators associated with community pharmacy PrEP delivery [23]. According to the COM‐B model, behaviour results from an interaction between capability, opportunity and motivation. Capability refers to an individual's psychological (knowledge) and physical (skills) ability to participate in an activity. Opportunity refers to external factors that can be environmental (physical) or social (societal influences) that make a behaviour possible, and motivation refers to the reflexive (beliefs, intentions) or automatic (emotion) cognitive processes that direct and inspire behaviour [23]. By examining the interplay of these factors relating to PrEP delivery, we can gain insights into the acceptability and feasibility of implementing community pharmacy PrEP delivery in the UK. This could inform the development of interventions to optimize PrEP delivery, thus advancing HIV prevention efforts and improving health outcomes.

The objective of this scoping review was to map and describe the existing evidence about the barriers to and facilitators of community pharmacy oral PrEP delivery for pharmacists and clients, according to the COM‐B Model.

## 2 METHODS

A scoping review was conducted following the methodological framework of scoping reviews [24], in accordance with the PRISMA (Preferred Reporting Items for Systematic Reviews and Meta Analyses) extension for scoping reviews [25]. We used the five‐stage scoping framework designed by Arksey and O'Malley [26] that involved (1) identifying the research question; (2) identifying relevant studies; (3) selecting studies; (4) charting the data; and (5) collating, summarizing and reporting the results.

*Stage 1*: The research question, “What is known from the previous literature about the barriers to and facilitators of community pharmacy PrEP delivery?” guided this review.
*Stage 2*: We used search terms related to HIV, PrEP and community pharmacies (see Table S1) to search five main bibliographic databases (MEDLINE [Ovid]; Embase [Ovid]; PsycINFO [Ovid]; CINAHL [EBSCOhost]; and the Cochrane Central Register of Controlled Trials [CENTRAL]) and five review databases (Cochrane Database of Systematic Reviews [CDSR]; Database of Promoting Health Effectiveness Reviews [DoPHER]; Epistemonikos; Health Evidence; and NIHR Health Technology Assessments) from inception to August 2023. The databases were searched in December 2022 and then an updated search was performed in August 2023. A manual search of the reference list of included literature and reviews related to the topic was also conducted to identify additional eligible research. Search results were imported into Endnote [27] and duplicates were removed. The final entries were then imported to Rayyan, a web‐based software program that facilitated screening [28] and three authors (JH and AS or CH) independently screened each title, abstract and full text according to the predefined inclusion/exclusion criteria (see below). Throughout the screening process, differences in opinions were resolved through discussion.
*Stage 3*: Literature was considered if it: (1) explored community pharmacy PrEP delivery; (2) included community pharmacists, PrEP clients, stakeholders, analysed data on PrEP initiation, continuation and adherence or explored community pharmacy PrEP interventions; (3) included studies about the encouragement of PrEP use (e.g. willingness, attitudes intentions); and (4) included information on the barriers to and facilitators of community pharmacy PrEP delivery. Trial registrations, protocols and news articles were excluded.
*Stage 4*: Data extraction was performed by CH, JH, JK, HF and SD and included author(s), year of publication, country, sample, study objective, methodology, and barriers and facilitators. A methodological quality assessment was not performed [29].
*Stage 5*: An overview of the volume and nature of the available evidence is represented graphically. The barriers to and facilitators of community pharmacy PrEP delivery are tabulated and synthesized according to the COM‐B model (and the context of studies identified) for pharmacists and clients.

## 3 RESULTS

**Figure 1:**
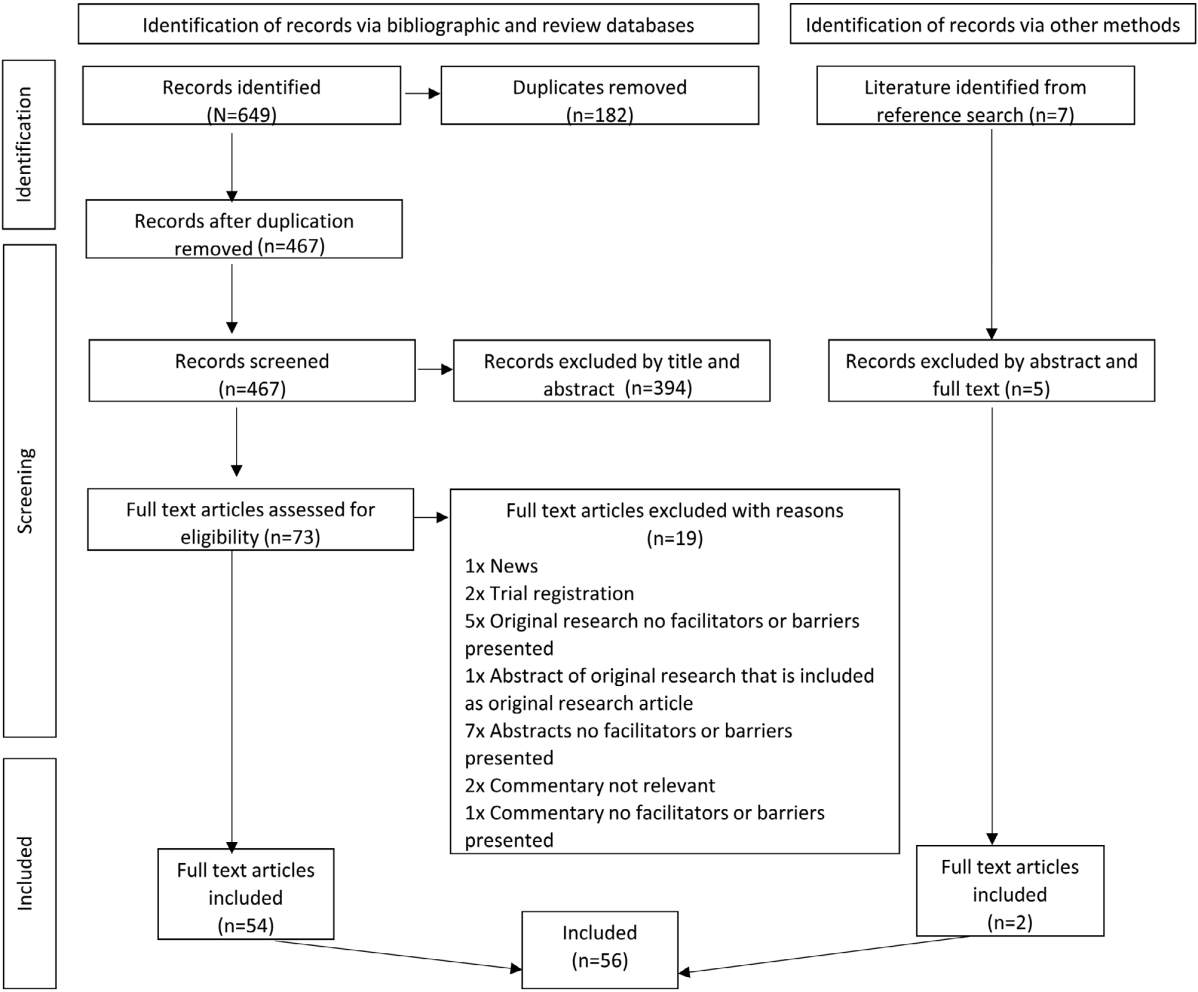
PRISMA flow diagram.

A total of 649 records were identified. After duplicate removal, 467 titles and abstracts were screened, resulting in the exclusion of 394 articles. The remaining 73 records underwent full‐text review, and 19 were excluded. From searching the references of the included studies, a further two studies were identified, resulting in a total of 56 records for inclusion (see Figure 1).

**Figure 2:**
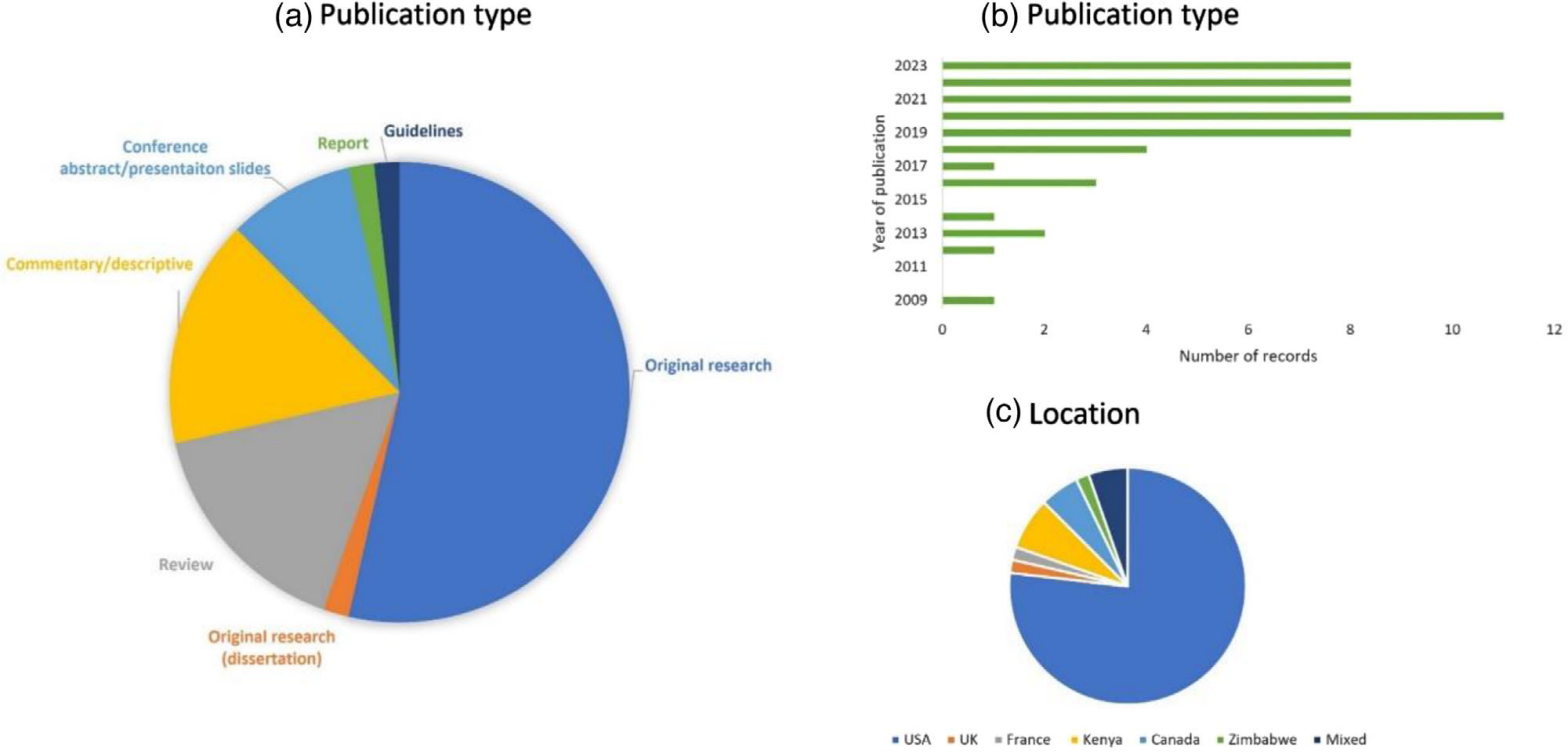
Characteristics of all the included records: Publication type (A), year of publication (B) and location (i.e. origin of study population or research) (C). Mixed location represents systematic reviews covering the literature from various countries.

Most of the included literature (Figure 2) was original research (55%), commentaries (16%) or reviews (16%), from the United States (77%), and was conducted during or after 2020 (63%). The methodological characteristics, study objectives, PrEP delivery/intervention explored and population, of the 56 included records, are summarized in Table S2.

A summary of the barriers and facilitators, according to the COM‐B model, is presented in Table 1 and synthesized descriptively below.

**Table 1:** Summary of the barriers and facilitators of community pharmacy PrEP delivery for pharmacists and clients

	Barriers		Facilitators	
COM‐B	Pharmacists	Clients	Pharmacists	Clients
Capability				
Psychological	Lack of PrEP knowledge and/or training [30−35]	Lack of PrEP awareness including the effectiveness of preventing HIV [30, 32, 36, 37]	Continued education/training for HIV/PrEP [33, 38−41]	Awareness/improving awareness of PrEP including the effectiveness of preventing HIV [30, 42, 43]
	Lack of knowledge/skills to deliver PrEP [33, 35, 38, 44−46]	Lack of awareness of payment assistance programmes [47]		
	Lack of knowledge about clients/not being able to identify potential clients [38, 39, 44, 48, 49]		Good knowledge of clients/being able to identify potential clients [32, 49]	
**Physical**	Insufficient familiarity with PrEP [38]	Concern that pharmacists lacked PrEP training [50]	Fewer (e.g. <10) years of pharmacy practice [39, 51, 52]	Knowing pharmacists had regulatory policies (e.g. quality standards) and oversight (audits) to adhere to [34]
	Concern about medical complications [53]		PharmD qualification (professional doctorate degree of pharmacy in the United States), fourth‐year pharmacy students [51, 52, 54, 56]	Knowing pharmacists had specific training/education for PrEP [34, 50]
	Less education (i.e. second‐year pharmacy students) [54]			
	Lack of PrEP guidelines [33]		Able to make PrEP recommendations, order lab tests [43, 57]	
	Lack of specialized PrEP delivery training/skills/experience [34, 35, 44−46, 58, 59]		Additional training including how to have sensitive conversations [31, 58−60]	
			Prior experience and familiarity of PrEP [47, 52, 54]	
**Opportunity**				
**Environmental**	Lack of skilled staff, staff turnover [31, 34, 44]		Hire staff/more staff on duty [34]	
	Economic instability [62]			
	Location and lack of transport [63]	Location of pharmacy [64, 65]	Convenient location/proximity [56, 59]	Pharmacies convenient location, accessibility and/or quick service [50, 53, 66, 67]
	Lack of staff time [31, 34, 35, 37, 53, 58, 68]	Perceptions of increased pharmacist workload [50]	Appointment systems [31, 37]	Attending specialized HIV service [66]
	Lack of physician oversight of PrEP initiation [61]			PrEP expert present and identifiable [69]
			Use of pre‐existing pathways/conversations [38, 53]	Use of existing pathways [70]
			Extended opening hours [39, 61, 67, 68]	Extended opening hours [39, 53, 66, 67]
				Living in an urban area [66]
	Lack of consultation room/privacy [34, 35, 37, 38, 53, 59, 61]	Lack of consultation room/space/privacy [32, 34, 55, 69]	Private consultation rooms/private space [31, 34, 44, 61]	Private space/confidentiality [32, 34, 69]
	Lack of facilities and/or systems [31, 33, 34, 44, 49, 53, 54]	Lack of pharmacy facilities [64, 65]	Shorter waiting lists/quick service [53, 56, 68]	Completion screening questionnaires/checklist prior to consultation/while waiting [71, 72]
			Provision of STI and HIV rapid tests [31]	Facility to self‐test for HIV [72]
			Allowing patients to test themselves for HIV [72]	Receiving HIV test results on the same day [71]
				Receiving ongoing monitoring at pharmacy [71]
				Able to complete online screening [71]
**Social**	Inability to advertise medication [44]		Permission to advertise PrEP [44]	
			Collaborative PrEP delivery service [31, 39, 58, 61, 67]	
	Inadequate screening and inconsistencies in workflow [73]		Clear referral process [73]	
			Advertising PrEP through a variety of different platforms [31, 32]	
**Motivation**				
**Reflective**	Perceived lack of need [31]	Perceived lack of need/decreased risk/lack of interest [32, 53, 64, 65]	Moral imperative/belief that PrEP would reduce HIV [31, 34, 56]	Perceived personal risk of HIV acquisition [74]
				Belief it would improve access [42]
	Belief that offering PrEP will increase sexually risky behaviours [54, 56, 62]	Belief that pharmacists were less knowledgeable about HIV drugs [50]		Interest in PrEP [56, 75]
	Belief that offering PrEP will increase STIs [41, 44, 46]	Belief that offering PreP will increase STIs [50]		Desire to be included in treatment decision‐making [71]
	Belief that offering PrEP will increase antiretroviral resistance rates [54, 58]		Belief that pharmacies are appropriate settings and highly trained [46, 53, 57, 61]	Belief that pharmacy is an acceptable place to initiate and receive PrEP [46]
	Belief that offering PrEP will reduce the use of other effective prevention methods [38]		In favour of community PrEP delivery/interest in PrEP [41, 56]	Fear of testing positive for HIV [72]
	Concern about PrEP adherence [58]	Concern of medication error [55]		Previous use of pharmacies/taken PrEP [55, 75]
	Concern about how pharmacists would handle drug toxicity/medical complications [53]			
	Affordability for clients [31, 32, 39, 44, 45, 49, 61, 62, 68, 76−78]	Cost of PrEP [22, 50]	Assistance with costs for clients [32, 34, 61]	Assistance with costs [22, 32, 66, 75]
	Affordability for pharmacists/lack of compensation [34, 35, 53, 58]		Costs reimbursed/PrEP seen as a source of profit [34, 53, 61, 64, 65]	
	Lack of clients asking for PrEP [39]		Being asked about PrEP [56]	
	Belief that specialist pharmacists should deliver PrEP [38, 56]	Pharmacies not considered a healthcare source [32]	Recognition of pharmacists as healthcare providers/well qualified/aligned with scope of work [35, 40, 61, 76]	
	Feeling uncomfortable counselling and prescribing PrEP [34, 39, 45, 47, 52, 53, 56, 68]		Feeling comfortable counselling [45, 56, 64]	
	Pharmacy provision may give clients a false sense of receiving medical treatment/important information may be missed [53]	Feeling uncomfortable seeking a pharmacist for PrEP [47, 50]	Comfortable performing point of contact testing for HIV [68]	Comfortable seeking a pharmacist for PrEP [50]
				Comfortable receiving HIV test result from pharmacist [79]
**Automatic**	Opposition from colleagues, subjective norms, perceived behavioural control and attitudes [38, 48, 80]		Belief about own capabilities and social influence [51]	Trust in pharmacists [32, 49, 70]
				Belief that pharmacists are well trained [42, 53]
	Stigma [32]	Stigma and misconceptions about PrEP [42, 81]	Stigma‐free environment and staff [31, 39, 49, 59, 67, 82]	Belief that pharmacy delivery would help decrease stigma [34, 39, 49]

## 4 SYNTHESIS OF RESULTS

### 4.1 Pharmacist‐level barriers and facilitators

#### 4.1.1 Capability

Barriers to PrEP delivery for pharmacists included a lack of awareness among pharmacists and clients of the legislation authorizing pharmacies in some states of America to provide PrEP [30, 36, 59]. Other barriers included a lack of knowledge, skills and training among pharmacists about the medication, and/or behaviour modification to reduce risks of acquiring HIV, prescribing PrEP and managing discussions about drug toxicity and/or medical complications [33, 35, 44−46, 53, 58]. Additionally, a lack of guidelines on the circumstances around PrEP initiation and continuation (e.g. whether clients who self‐test for HIV can be supplied PrEP) [33], and a lack of clarity or knowledge about which clients would benefit from PrEP were reported to be barriers to identifying and counselling those at risk of acquiring HIV [38, 39, 44, 48, 49].

Facilitators included pharmacists in the United States having a good PrEP knowledge, able to identify who would benefit from PrEP [32, 49] and receiving continuing education or specific HIV/PrEP training [33, 38−41]. Additional facilitators were pharmacists having the authority to make recommendations about PrEP safety, efficacy and acquisition [57], having a doctorate degree of pharmacy (PharmD) [51, 52, 54, 56], having fewer years of pharmacy practice experience and, therefore, having public health content during their education [39, 51, 52]. Prior familiarity of PrEP guidelines and subsequent confidence in prescribing PrEP [47, 52, 54] were also facilitators.

#### 4.1.2 Opportunity

Economic and social opportunity barriers for pharmacists included the country's economic instability [62] and the inability to advertise branded medications in pharmacies [44]. For the more specific aspects of PrEP delivery in community pharmacies, such as PrEP counselling and prescribing, pharmacists in the United States and Kenya reported a high turnover of staff [72] and the lack of skilled staff (e.g. only having one pharmacist on duty per shift) as barriers [31, 33, 34]. Similarly, lack of staff time [31, 34, 35, 37, 53, 58, 68] was also found to be a barrier, particularly when needing longer consultation times [31] to initiate clients on PrEP [72] and carry out the required monitoring (e.g. kidney function) [83]. There was also concern that an increased workload would negatively impact the time allocated to delivery of other services [43]. Pharmacists also identified physical barriers, such as the absence of private spaces or consultation rooms [33−35, 38, 53, 61], and laboratory facilities to carry out, process and record STI, HIV and kidney screening and monitoring [31, 33, 34, 44, 49, 53, 54].

Facilitators included community pharmacies being perceived as ideal locations for PrEP delivery, accessible, conveniently located and with extended opening hours [33, 35, 39, 47, 53, 56, 67, 68]. To overcome the barriers of staff capacity and time, pharmacists suggested using appointment systems to schedule PrEP screening [31, 37], hiring more staff or having more staff on duty per shift to conduct PrEP counselling and dispensing [34]. Pharmacists working in pharmacies with a larger number of full‐time staff were found to be more comfortable and able to spend the necessary amount of time counselling clients about PrEP [56]. Pharmacists also suggested that PrEP delivery could be facilitated by incorporating it into pre‐existing services or pathways (e.g. STI testing) [38, 53].

Having a private consultation room [31, 34, 44, 61] to have conduct sensitive conversations, in addition to facilities to enable STI and HIV testing [31], were reported to be facilitative of pharmacy PrEP delivery. If there was a lack of facilities, pharmacists reported that allowing clients to self‐test themselves for HIV [72] or having a collaborative service with other healthcare providers could help facilitate PrEP delivery, adherence and continuation [31, 39, 58, 61, 67]. Having a clear referral process for pharmacists to provide clients with the necessary treatment options for conditions identified during PrEP delivery was also found to facilitate PrEP delivery [73].

#### 4.1.3 Motivation

Improving PrEP accessibility via community pharmacies was not judged to be a moral imperative by some pharmacists because pharmacy clients were not perceived to be at risk of acquiring HIV/HIV becuase rates were assumed to be low [31]. Other motivational barriers included experiencing opposition to PrEP delivery from colleagues [38, 48, 80], and concerns that increasing accessibility to PrEP would increase risky sexual behaviours [54, 56, 62], STIs [41, 44, 46], antiretroviral resistance [54, 58] and negatively impact the use of other available cost‐effective STI preventative methods (e.g. condoms, abstinence) [38].

Pharmacists in the United States and Zimbabwe reported the affordability of PrEP, and whether medical insurance would cover the costs of counselling and prescribing [31, 32, 39, 44, 45, 49, 61, 62, 68, 76−78] to be significant barriers to PrEP delivery. Pharmacists were concerned that they (or the pharmacy business they worked for) would not profit or be adequately compensated for these services [34, 35, 53, 58]. Pharmacists also reported concern about the stigma people could experience from community members when accessing PrEP [32], in addition to reporting a sense of discomfort discussing sexual health/histories, positive HIV test results, and counselling clients on behaviour modification and PrEP effectiveness [34, 45, 47, 52, 53, 56, 68, 69]. Some pharmacists also reported that they were reluctant to deliver PrEP without clients having had a prior medical consultation [61], and that PrEP delivery should only be done by pharmacists specializing in HIV [56].

Being in favour of pharmacy PrEP delivery [41, 56], believing that pharmacies were appropriate settings to deliver PrEP [46, 53, 57, 61, 76] and that pharmacists were healthcare providers [35, 40, 76] who were well qualified/trained were found to facilitate PrEP delivery. Other motivational facilitators included having an interest in HIV prevention [84], believing that PrEP could reduce new cases of HIV [31, 34, 56], feeling comfortable counselling about PrEP and HIV risk reduction behaviours [45, 56, 64] and performing point of contact testing [68].

To improve PrEP initiation, continuation and adherence, pharmacists reported that facilitating free access to PrEP or ensuring assistance with the cost [32, 34, 61] would be facilitative, particularly in the United States and for those most in need. Pharmacists also reported that the financial costs of PrEP to pharmacists would need to be reimbursed or covered by insurance companies [34, 53, 61, 64, 65] so that PrEP delivery was a source of income [34].

An additional facilitator of PrEP delivery for pharmacists was the belief that pharmacists should be able to provide a stigma‐free environment [31, 33, 39, 49, 67], that was culturally appropriate and convenient in which to have sensitive conversations [82].

### 4.2 Client‐level barriers and facilitators

#### 4.2.1 Capability

For pharmacy clients, barriers to PrEP delivery via community pharmacies were reported to be a lack of PrEP awareness, including its effectiveness [30, 32, 36, 37]. Clients also reported hesitancy towards pharmacy PrEP delivery because they lacked knowledge of pharmacists’ HIV and/or PrEP specialized knowledge/training [50].

Improving client awareness of PrEP was reported to facilitate PrEP delivery [42, 43, 50]. Clients reported that the acceptability of community pharmacy PrEP delivery would be further facilitated if they were made aware of the regulatory policies (e.g. quality standards), and oversight (audits) that pharmacists had to adhere to [34], and any additional training or education for PrEP delivery that pharmacists had obtained [34, 50].

#### 4.2.2 Opportunity

Actual or perceived environmental barriers to PrEP delivery in community pharmacy for clients included the lack of private consultation rooms [34, 55] or space to ensure privacy and confidentiality [32, 69]. The location of the pharmacy [65] was also reported to be a barrier, particularly for clients relocating [64]. Clients also recognized the lack of pharmacy facilities for laboratory testing [42] and pharmacist workloads as barriers [50].

Clients perceived the accessibility of community pharmacies in terms of location, speed of service and extended opening hours [39, 50, 53, 66, 67] to facilitate PrEP delivery, particularly if living in an urban area [66]. Other important facilitators for clients were the presence of a private space to discuss and deliver PrEP to ensure confidentiality and respect [32, 34, 69], offering PrEP alongside other existing care pathways and services (e.g. opiate substitution therapy) and the presence of a pharmacist specializing in HIV service provision who could be easily identified (e.g. by their clothing) as the person to speak to about PrEP [69]. Additional facilitators for clients included the pharmacies being able to provide HIV test results on the same day, offering ongoing monitoring for adherence support and risk reduction and being able to complete screening questionnaires prior to the consultation [71].

#### 4.2.3 Motivation

The US clients were concerned about the cost of PrEP and pharmacists being unable to process their insurance [22, 50]. Clients also reported concern that increasing access to PrEP could result in an increase in STIs [50]. Additional barriers for clients were believing that there was a general and/or personal lack of need for PrEP [32, 53, 64, 65], believing that pharmacists were not healthcare providers [32] and were not knowledgeable about HIV drugs [50] and that pharmacy PrEP delivery could result in medical errors [55]. Clients also reported feeling less comfortable seeking a pharmacist for PrEP information and delivery [47, 50], preferring to speak to their physician [50], or organizations that cater to the lesbian, gay, bisexual, transgender, queer/questioning and other (LGBTQ+) community or organizations that help cover the costs of PrEP [47]. Some clients were also hesitant to be screened in pharmacies for fear of testing HIV positive [72]. Clients were also concerned about the misconceptions of PrEP being a medication for people living with HIV and that they could experience stigma from pharmacists [42, 81].

Clients’ motivation to seek PrEP from community pharmacists was facilitated by an interest in PrEP [56, 75], a desire to be involved in treatment decisions [71], a belief that pharmacy PrEP delivery would improve access to PrEP [32, 49, 70], feeling comfortable seeking PrEP information and prescriptions from pharmacists [50], receiving HIV test results from pharmacists [46, 53] and having trust in pharmacists [34, 39, 49]. Clients who perceived themselves to be at a greater risk of acquiring HIV due to engagement in condomless sex or sex with multiple partners were also found to be more willing to be screened in community pharmacies. This was compared to those who did not engage in condomless sex, and those who perceived themselves to be at lower risk of acquiring HIV [74].

Believing that pharmacists were well trained to deliver PrEP [53] and that pharmacies were acceptable places to initiate and receive PrEP was also found to facilitate PrEP delivery [42, 46, 53]. Other facilitators included PrEP being available free or at a low cost to clients [22, 32, 66, 72, 75], having insurance to cover the costs of PrEP [74] and believing that PrEP delivery in community pharmacies could circumvent the stigma associated with PrEP and HIV, in part by offering clients the opportunity to obtain PrEP discretely [34, 39, 49].

## 5 DISCUSSION

In this scoping review, we mapped the barriers and facilitators of community pharmacy PrEP delivery to the COM‐B model. The current review highlights the increase in research in this area, particularly in the United States, after pharmacists in certain states were authorized to prescribe PrEP or deliver PrEP under a collaborative practice agreement. While there have been systematic and scoping reviews on pharmacy PrEP delivery [35, 85], none have aimed to identify and map the potential barriers and facilitators of PrEP delivery, according to a behavioural theory or model. Subsequently, the findings from the current review provide important insight for the development of interventions to optimize community pharmacy PrEP delivery. In this review, barriers identified included lack of PrEP awareness, familiarity knowledge, skills and training (capability), lack of staff capacity, time [31, 34, 35, 37, 44, 53, 58, 68] and pharmacy facilities (opportunity), the financial cost of PrEP to pharmacists and clients and the belief that PrEP delivery could lead to risky behaviours and higher rates of STIs (motivation). Facilitators identified included improving client and pharmacist awareness of PrEP, the provision of PrEP training and education (capability), having an appointment system, using pre‐existing pathways or services, the accessibility of community pharmacies (opportunity) and the belief that PrEP delivery could be a source of income or profit and prevent new cases of HIV (motivation).

In translating the findings to UK community pharmacy PrEP delivery, some of the opportunity barriers presented have already been addressed. The vision for community pharmacy is to provide more clinical services, to support and manage demands on the NHS [86]. Consequently, most UK pharmacies have private consultation rooms and, in some regions, there are well‐established links with other healthcare providers (e.g. sexual health clinics) for the provision of services. For example, the emergency contraception referral pathway is supported by regular training and collaboration with local pharmaceutical committees. Some of these services could potentially be expanded to include the provision of PrEP, particularly the reproductive and sexual health services that are requested most frequently (e.g. condoms, STI self‐testing kits, emergency contraception) [87]. Patient group directions which authorize pharmacy professionals to supply particular medications to clients presenting in person also offer potential opportunities for PrEP delivery.

Other barriers identified, such as the lack of skilled staff/staff capacity, highlight the increasing workload of pharmacists [17, 18, 88] and the potential lack of feasibility of introducing PrEP delivery to community pharmacies [89]. Although hiring more staff was identified to help overcome staff capacity barriers, results from the Pressures Survey conducted by the Pharmaceutical Services Negotiating Committee found that 71% of community pharmacies were experiencing a shortage of pharmacists [90], suggesting a lack of pharmacists to employ even though there is demand. A better understanding of issues facing pharmacists in the UK could improve awareness and inform future investment in education and training in addition to effective workplace planning for pharmacy service provision, including PrEP delivery.

The accessibility of pharmacies, as highlighted in this review, could offer an easy, less intimidating environment from which to access PrEP. This may be particularly important for individuals with lower levels of literacy who find the increasing use of digital technology required to access health services challenging [91]. Community pharmacy PrEP delivery could, therefore, help to reduce inequalities among those who are disproportionately affected by HIV, in part because ease of access has been shown to be key to the uptake and maintenance of PrEP [85, 92]. Similarly, addressing the motivational barriers identified by pharmacists and clients could reduce stigma, facilitate awareness of LGBTQ+ issues and improve client confidence in pharmacists’ medical expertise and capability to provide services. Future research needs to examine the perceived acceptability and feasibility of UK pharmacies for PrEP delivery, particularly among pharmacists and individuals at elevated risk of acquiring HIV who are not currently accessing PrEP from sexual health clinics. This research should also explore whether the stigmatizing attitudes of some pharmacists could act as barriers to service provision [17].

Overall, the results suggest that to change behaviour and facilitate community pharmacy PrEP delivery in the UK, pharmacists and clients should be provided with a training intervention that fosters beliefs about the positive impact of PrEP delivery, stimulates and harnesses client's interest and provides financial support for pharmacists and clients to enhance motivation for PrEP delivery, initiation, continuation and adherence. However, the issue of high drug procurement costs for community pharmacies may be a significant barrier for PrEP delivery in the UK, particularly if pharmacy PrEP delivery is to provide PrEP free of charge to clients. PrEP delivery via pharmacies may be unlikely until procurement costs have been levelled across providers, even if the barriers and facilitators identified in the review are addressed and leveraged.

There are limitations to our review. Most of the research reviewed was from the United States so the barriers and facilitators presented may be specific to this population (e.g. payment for healthcare and differing contractual frameworks). More work is needed to understand UK‐specific barriers and facilitators. Further, several studies included pharmacists working in settings other than community pharmacies (e.g. General Practitioner (GP) and hospitals). This made the findings specific to community pharmacists hard to distinguish. Nonetheless, the current scoping review has methodological strengths (i.e. searching of multiple databases, handsearching of citations) and employs the COM‐B model as a framework to synthesize the evidence.

## 6 CONCLUSIONS

This scoping review provides valuable insights into the barriers and facilitators of PrEP delivery in community pharmacies. It highlights the multifaceted nature of PrEP delivery and underscores the need to consider individual external and reflective factors influencing the behaviour of pharmacists and clients in providing and accessing PrEP services. This review is the first step towards developing theory and an acceptable, effective evidence‐based intervention for UK community pharmacies. By considering all aspects of the COM‐B, community pharmacies in the UK could become crucial players in expanding PrEP accessibility and uptake, contributing significantly to HIV prevention efforts.

## COMPETING INTERESTS

The authors declare no competing interests.

## AUTHORSʼ CONTRIBUTIONS

CH, JK, SD, HF, JS, CB, JC, LH, SC and JH contributed to the research planning. AS and SD contributed to the literature search, AS, JH and CH to study selection, and CH, JH, JK, HF and SD to data extraction. CH and JH drafted all versions of the manuscript, and all authors contributed to and approved the final version for publication.

## FUNDING

This research was funded by Gilead Sciences, Inc, and supported by the National Institute for Health and Care Research Applied Research Collaboration West (NIHR ARC West) and the National Institute for Health and Care Research Health Protection Research Unit (HPRU) in Behavioural Science and Evaluation at the University of Bristol, and in Sexually Transmitted and Blood Borne Infections at UCL, both in partnership with UKHSA.

## DISCLAIMER

The views expressed in this article are those of the authors and not necessarily those of the NIHR, UKHSA or the Department of Health and Social Care.

## Supporting information


**Table S1**. Search terms and strategies used for the five main bibliographic databases and five review databases

**Table S2**. Summary of the total (*N* = 56) included literature, methodological characteristics, study objectives and population

## Data Availability

Data are available upon reasonable request from the corresponding author.
