# Supplementary material for: Facilitators and barriers to community pharmacy PrEP delivery: a scoping review
Source: J Int AIDS Soc. 2024 Mar 17;27(3):e26232. doi: 10.1002/jia2.26232 (PMC10945033; doi:10.1002/jia2.26232)
Supplement: Supplementary file 2 — Table S2. Summary of the total (N = 56) included literature, methodological characteristics, study objectives and population [file JIA2-27-e26232-s002.docx]

Supplementary Table 2

*Summary of the total (N=56) included literature, the methodological characteristics, study objectives and population*

| **Author** | **Year** | **Location** | **Publication type** | **Study design** | **Study objective** | **PrEP delivery/ Intervention explored** | **Study population** |
| --- | --- | --- | --- | --- | --- | --- | --- |
| Alohan et al (74) | 2023 | USA | Original research | Cross sectional survey | To examine the correlates of patient (men-who-have-sex-with-men, MSM) willingness to be screened for PrEP in pharmacies | Pharmacy PrEP screening (sexual behaviour history STI and HIV screening) . | Clients (MSM) |
| Bellman et al.(31) | 2022 | USA | Original research | Cross sectional survey and interviews | To access implementation of PrEP in San Francisco pharmacies and barriers and successes of implementation | Pharmacy PrEP initiation via pharmacy with a collaborative practice agreement. | Pharmacists |
| Bouetard & Cordel (41) | 2021 | France | Conference abstract | Cross sectional survey | To measure community pharmacists' knowledge, experience and perception regarding PrEP | Pharmacy promote PrEP adherence. | Pharmacists |
| Broekhuis et al. (57) | 2018 | USA | Original research | Cross sectional survey | To characterise pharmacists' knowledge about and willingness to provide PrEP | Pharmacy PrEP delivery via a collaborative practice agreement.  PrEP initiation. | Pharmacists |
| Booker et al. (43) | 2023 | USA | Original research | Cross sectional survey and interviews | To determine pharmacists’ acceptance of a pharmacist PrEP prescribing service | Pharmacy PrEP initiation and continuation) and laboratory monitoring (STI, HIV, Kidney & liver function). | Pharmacists |
| Bruno & Saberi (59) | 2012 | USA | Commentary |  | To discuss reasons for a protocol-based, clinical pharmacist-run PrEP clinic. | Pharmacy PrEP initiation and continuation and STI and HIV screening, counselling on safe sex. | N/A* |
| Burns et al. (83) | 2023 | USA | Original research | Cross sectional survey | To determine the perceived feasibility and acceptability of prescribing PrEP by pharmacists | Pharmacy PrEP prescribing. | Pharmacists* |
| Cernasev et al. (32) | 2022 | USA | Original research | Focus groups | To characterise Tennessee pharmacists' perceptions about access to PrEP | Various models of pharmacy PrEP delivery to improve access, initiation, and continuation of PrEP (e.g., education).  . | Pharmacists |
| Cernasev et al. (73) | 2023 | Only studies conducted in the USA | Review | Systematic review | To explore the tole of pharmacists, pharmacy services and interpersonal collaborations for persons seeking PrEP | Pharmacy PrEP screening and initiation via collaborative practice agreements. |  |
| Clauson et al. (56) | 2009 | USA | Commentary | N/A | To discuss reasons for a protocol-based, pharmacist-run PrEP clinic. | Pharmacy PrEP screening, education, and management (e.g., drug interactions). | N/A |
| Crawford et al. (75) | 2021 | USA | Original research | Cross sectional survey | To examine the correlates of willingness to discuss PrEP with pharmacy staff and screen for PrEP in a pharmacy setting. MSM. | Pharmacy PrEP screening, initiation and STI and HIV screening. | Clients (MSM) |
| Crawford et al. (46) | 2021 | Only studies conducted in USA | Review | Systematic literature review | To synthesise the recent literature on the role of pharmacists across the HIV prevention and care continuums | Pharmacy PrEP delivery (STI and HIV screening, counselling, dispensing) independently and via collaborative practice agreements. |  |
| Crawford et al. (47) | 2020 | USA | Original research | Qualitative interviews | To understand the perceptions and support for pharmacy-based pre-exposure prophylaxis (PrEP) delivery among pharmacists and MSM | Pharmacy PrEP delivery (STI and HIV screening, PrEP dispensing)  Pharmacy PrEP initiation, independently and via collaborative practice agreements. | Pharmacists and Clients (MSM) |
| Crawford et al. (68) | 2022 | USA | Original research | Pilot of protocol | To measure feasibility, acceptance, safety, and PrEP use at baseline and at 3-months. | Pharmacy PrEP STI and HIV screening and dispensing. | Clients (MSM) |
| Dong et al. (82) | 2019 | USA | Opinion | N/A | To respond to Havens et al 2019 study (below) and provide an alternative perspective, with examples of how PrEP has been successfully implemented in pharmacy settings. | Pharmacy PrEP initiation, STI and HIV screening. | N/A |
| Farmer et al. (39) | 2019 | USA | Review | Literature review | To summarise evidence on pharmacist involvement in different models of care providing PrEP services and to identify opportunities to maximise and expand the role of the pharmacist to improve access to PrEP. | Various models of pharmacy PrEP delivery (e.g., STI and HIV screening, counselling, PrEP dispensing, prescribing). |  |
| Garrison et al. (62) | 2021 | USA | Review | Narrative review | To summarise interventions (2017-2020) aimed at improving PrEP uptake, adherence and persistence of PrEP. | Various models of pharmacy PrEP delivery (e.g., counselling, PrEP initiation and monitoring). |  |
| Gregory (93) | 2020 | USA | Commentary | N/A | To report on the new law in California that allows pharmacists to provide PrEP. | Pharmacy PrEP initiation, STI and HIV screening. | N/A |
| Griffith et al. (76) | 2022 | USA | Commentary | USA | To discuss access and adherence to PrEP in community pharmacy? | Pharmacy PrEP awareness raising, signposting and PrEP initiation and  Continuation. | N/A |
| Goswami (71) | 2022 | USA | Original research (PhD dissertation) | Survey and discreet choice experiment | To elicit preferences for attributes of a community pharmacy-based PrEP delivery program. | Hypothetical pharmacy PrEP delivery (STI and HIV screening, counselling, dispensing, monitoring). | Clients (MSM) |
| Havens et al.(66) | 2019 | USA | Original research | Quantitative analysis of use of service/feasibility trial (pilot) | To assess the acceptability and feasibility of a pharmacist-led HIV screening and PrEP program (P-PrEP) for individuals at risk for HIV acquisition. | Pharmacy PrEP initiation, continuation, STI and HIV screening via collaborative practice agreement . | Clients |
| Hazen et al. (64) | 2017 | USA | Conference abstract | Retrospective cohort | To characterise PrEP medication utilisation patterns and factors associated with medication adherence. | Unclear. | Clients |
| Hopkins et al.(37) | 2021 | USA | Original research | Qualitative Interviews | To assess pharmacists’ and pharmacy technicians’ perspectives regarding the implementation of PrEP screening and dispensing. | Pharmacy STI and HIV screening, PrEP dispensing, | Pharmacy technicians and pharmacists |
| Huang et al (94) | 2022 | USA | Original research | Cross sectional analysis of national pharmacy database | To better understand PrEP cessation. | Pharmacy PrEP prescriptions and continuation. | Pharmacy database |
| Hughes et al. (77) | 2019 | Canada | Guidelines | N/A | To provide an overview of guidelines and highlight the role pharmacists can have in HIV prevention. | Pharmacy PrEP screening, initiation and continuation, providing PrEP information and risk reduction counselling, assisting with medication procurement and provision. | N/A |
| Josma et al. (79) | 2023 | USA | Original research | Qualitative Interviews | To examine black men-who-have-sex-with-men (BMSM) beliefs about accessing PrEP in pharmacies. | Pharmacy PrEP initiation, STI and HIV screening and dispensing via collaborative practice agreement. | Clients (BMSM) |
| Kazi et al (22) | 2019 | USA | Commentary |  | To discuss pharmacy led PrEP delivery. | Various models of pharmacy PrEP delivery. |  |
| Kennedy et al. (35) | 2022 | Mixed | Review | Systematic review | To evaluate the evidence for distributing PrEP through pharmacies. | Pharmacy PrEP initiation and continuation, STI, HIV screening, kidney monitoring delivery via a collaborative practice agreement where pharmacists operated under physician oversight.  . | Pharmacists and people interested in PrEP |
| Khosropour et al. (61) | 2020 | USA | Original research | Pilot study | Pilot to facilitate PrEP uptake and decrease time to PrEP initiation. | Pharmacy PrEP initiation prior to clinician kidney and Hepatitis B screening within six weeks.  . | Clients |
| Khosropour et al. (81) | 2023 | USA | Original research | Mixed methods evaluation | To describe PrEP initiation and persistence of individuals who participated in a community pharmacy PrEP delivery program and to examine the barriers motivations and facilitators to PrEP initiation and persistence. | Pharmacy PrEP initiation and continuation prior to clinician kidney and Hepatitis B screening within six weeks. | Clients |
| Koester et al. (53) | 2020 | USA | Original research | Qualitative case study | To assess attitudes among key stakeholders about a California policy to allow community pharmacists to deliver HIV PrEP. | Pharmacy PrEP prescribing, dispensing, STI and HIV screening. | Pharmacists and stakeholders |
| Lopez et al. (30) | 2020 | USA | Commentary | N/A | To encourage key stakeholders to advocate for implementing community pharmacy–initiated PrEP and provide recommendations for implementing PrEP in a community pharmacy. | Pharmacy PrEP initiation STI and HIV screening via collaborative practice agreement. | N/A |
| Lutz et al. (50) | 2021 | USA | Original research | Cross sectional survey | To assess patient perspectives of pharmacist PrEP prescribing and identify potential barriers to acceptance of pharmacy prescribed PrEP. | Pharmacy PrEP prescribing. | Patients receiving antiretroviral medications |
| MacDonald et al. (42) | 2023 | USA | Original research | Cross sectional survey and interviews | To determine target users’ acceptance of a PrEP prescribing service by pharmacists. | Pharmacy STI and HIV screening, and education PrEP prescribing. | Clients |
| Matyanga et al. (60) | 2014 | Zimbabwe | Original research | Cross sectional survey | To assess pharmacists’ knowledge, perception and willingness to provide PrEP. | Pharmacy PrEP dispensing. | Pharmacists |
| Mayers et al. (67) | 2018 | USA | Review | Critical review | To analyse the current state of PrEP implementation in the US by reviewing barriers and innovative solutions to enhance PrEP access and uptake. | Various models of pharmacy PrEP delivery (e.g., STI, HIV screening, counselling, dispensing, prescribing). |  |
| McCree et al. (65) | 2020 | USA | Report |  | To discuss potential roles that pharmacists and pharmacies can play in delivering PrEP. | Various models of pharmacy PrEP delivery (e.g., collaborative practice agreement, STI and HIV screening, prescribing. | N/A |
| Meyerson et al.(55) | 2019 | USA | Original research | Cross sectional survey | To identify factors associated with PrEP dispensing and comfort with PrEP counselling among community pharmacists to inform the design of evidence-based pharmacy-practice interventions. | Pharmacy counselling patients and dispensing PrEP. | Pharmacists (managing pharmacists) |
| Myers et al. (40) | 2019 | USA | Commentary |  | To share recommendations on community pharmacy PrEP delivery | Various models of pharmacy PrEP delivery (e.g., STI and HIV screening). | N/A |
| Nakambale et al. (72) | 2023 | Kenya | Original research | Observations | To identify early implementation barriers of pharmacy-based PrEP delivery and actions that pharmacy providers took to help address these. | Pharmacy PrEP initiation and continuation under remote clinical oversight for a fee. HIV and STI screening, kidney function Counselling. | Pharmacists and Clients |
| Okoro & Hillman (44) | 2018 | USA | Original research | Cross sectional survey | To assess knowledge and experience, describe perceptions and attitudes and identify training needs of community-based pharmacists. | Pharmacist PrEP delivery (e.g., counselling, STI and HIV screening, collaborative PrEP prescribing). | Pharmacists |
| Ortblad et al. (33) | 2020 | Kenya | Original research | Stakeholder consultation* | To develop a pathway for pharmacy-based PrEP delivery in Kenya. | Pharmacy PrEP prescribing, dispensing counselling, HIV screening. | PrEP stakeholders |
| Ortblad et al. (58) | 2020 | Kenya | Commentary |  | To discuss the adoption of pharmacy- based PrEP delivery in Africa in relation to the USA. | Pharmacy PrEP prescribing, dispensing counselling, HIV screening,). | N/A |
| Przybyla et al.(54) | 2019 | USA | Original research | Cross sectional survey | To assess pharmacy students' awareness, knowledge and perceptions towards HIV, PrEP, confidence and intentions to counsel customers on PrEP and preferred PrEP training. | Pharmacy PrEP counselling. | Pharmacy students enrolled in a Doctor of Pharmacy (PharmD) |
| Roche et al.(34) | 2021 | Kenya | Original research | Qualitative Interviews | To identify factors that may influence the implementation of pharmacy-based PrEP delivery in Kenya, to inform the design and implementation of a pharmacy PrEP care pathway. | Pharmacy STI and HIV screening and prescribing PrEP. | pharmacy clients. pharmacy providers, PrEP clients, PrEP providers |
| Rousseau et al. (49) | 2021 | Mixed | Review |  | To summarise novel platforms for HIV prevention outside of the traditional health facilities environment. | Pharmacy PrEP delivery through collaboration with nurse practitioners and physician assistants . |  |
| Sawkin et al. (84) | 2016 | USA | Conference abstract | Unclear | To develop and test a pharmacy led clinic to expand access to PrEP. | Unclear | Unclear |
| Shaeer et al. (45) | 2013 | USA | Conference abstract | Cross sectional survey | To assess pharmacists experience with, knowledge of, and perceptions about HIV PrEP and identify areas for pharmacist training. | Unclear. | Pharmacists |
| Smith et al. (69) | 2021 | UK | Original research | Qualitative Interviews | To explore views of people who inject drugs (PWID) who might benefit from PrEP provision and service providers working with PWID to understand willingness to use PrEP and literacy of PrEP, contributing to the development of a PrEP service. | Pharmacy STI and HIV screening, monitoring ongoing usage and dispensing PrEP. | Clients (PWID) |
| Tung et al. (63) | 2019 | USA | Original research | Service evaluation | To describe the service (pharmacist managed PrEP clinic in a community pharmacy setting) and report initial experiences. | Pharmacy PrEP initiation and continuation and management under the supervision of a physician medical director.  . | Pharmacists and clients |
| Unni et al. (51) | 2016 | USA | Original research | Cross sectional survey | To measure pharmacists' knowledge about PrEP, perceptions about their PrEP knowledge and intentions to counsel customers about PrEP. | Pharmacy PrEP counselling, | Pharmacists |
| Wilby et al. (52) | 2020 | Mixed | Review | Narrative review | To identify priority areas and key gaps for continuing professional development (CPD) needs relating to PrEP for practicing pharmacists. | Pharmacy STI and HIV screening, PrEP initiation. |  |
| Yoong et al. (48) | 2016 | Canada | Original research | Cross sectional survey | Evaluate pharmacists' attitudes and readiness for PrEP. | PrEP education. | Pharmacists |
| Yoong et al. (38) | 2013 | Canada | Conference abstract | Unclear | To determine Canadian pharmacists' enthusiasm for PrEP and their opinions on PrEP implementation. | PrEP education. | Pharmacists |
| Zhao et al. (85) | 2022 | USA | Review | Scoping review | To explore pharmacy-based initiatives to increase PrEP use. | Various models of pharmacy PrEP delivery. |  |
| Zhu et al. (70) | 2020 | USA | Original research | Cross sectional survey | To determine customers perceptions of pharmacist-prescribed PrEP, determining whether perceptions differed between customers who had previously been prescribed PrEP and those who had not and those who had at least one indication for PrEP verses those who did not. To identify what customer characteristics, make customers more willing to seek PrEP from pharmacies. | Pharmacy PrEP prescribing. | Clients |

*Note*: *Record does not include or refer to community-based pharmacists only, clinical pharmacists or other health care professionals are included or referred to. STI= sexually transmitted infection. HIV= Human immunodeficiency virus. PWID = People who inject drugs.
